# Thermoformability of Biopolymer Composites with Coffee Silverskin

**DOI:** 10.3390/polym17223067

**Published:** 2025-11-19

**Authors:** Ana C. Machado, Mariana Beltrão, Maria C. R. Castro, Carla I. Martins, Vasco Cruz, Pedro V. Rodrigues, Fernando M. Duarte

**Affiliations:** IPC—Institute for Polymers and Composites, University of Minho, 4804-533 Guimarães, Portugal; d12017@dep.uminho.pt (M.B.); cidaliacastro@dep.uminho.pt (M.C.R.C.); cmartins@dep.uminho.pt (C.I.M.); vasco.cruz@dep.uminho.pt (V.C.); pedro.rodrigues@dep.uminho.pt (P.V.R.); fduarte@dep.uminho.pt (F.M.D.)

**Keywords:** coffee silverskin, thermoforming, sustainable packaging

## Abstract

The valorisation of agro-industrial residues in polymer composites represents a promising strategy for waste valorisation and the development of sustainable packaging materials. In this study, coffee silverskin (CSS), a lignocellulosic by-product, was added at concentrations up to 15 wt.% and processed into sheets via extrusion, followed by thermoforming using moulds with different draw ratios. Processability (MFI) and structural (FTIR), morphological (SEM, optical microscopy), thermal (TGA, DSC), and mechanical characterizations (tensile tests) were performed. Although the SEM images showed that CSS particles were well dispersed in the polymer matrix, and the mechanical behaviour was negatively affected when compared to the neat biopolymer. On the other hand, the addition of CSS increased the melt flow index, suggesting a lubricating/plasticizing effect. DSC results showed a reduction in cold crystallization temperature with CSS addition, confirming a nucleating effect, while glass transition and melting temperatures remained unchanged. Despite a narrower thermoforming temperature window with increasing CSS content, defect-free parts with adequate mould replication were successfully obtained for all formulations. Overall, the incorporation of CSS into PLA matrix provides a viable pathway for producing thermoformable as potential compostable composites, enabling waste valorisation within a circular bioeconomy framework.

## 1. Introduction

In recent years, the incorporation of natural fillers into biopolymers has emerged as a promising strategy for developing sustainable materials. Diverse plant-based materials including lignocellulosic fibres (sisal, hemp, flax, bamboo), starch, cellulose derivatives and agro-industrial by-products have shown remarkable success when blended into polymer matrices such as polylactic acid (PLA), polyhydroxyalkanoates (PHA), and polybutylene succinate (PBS). Beyond improving mechanical strength, the addition of fillers also reduces production costs and improves the biodegradability of the materials [1,2].

Despite the growing interest, the production of composite sheets suitable for thermoforming remains technically challenging. The addition of natural fibres typically increases melt viscosity and shear stress due to fibre networking and polymer-fibre interactions. These effects restrict the melt flow and difficult the formation of a uniform and defect-free sheets [3]. Furthermore, fibre reinforcement generally enhances stiffness but reduces elongation at break and ductility, thereby limiting the drawability required for complex thermoformed shapes, increasing the risk of cracking or tearing [1]. Poor interfacial adhesion between hydrophilic natural fibres and hydrophobic polymer matrices creates additional problems by promoting stress concentration points and void formation, compromising the mechanical integrity of the formed sheets. To address this, chemical modifications of fibres are often employed to improve fibre-matrix compatibility [4,5]. Moreover, natural fillers also influence crystal morphology and crystallization kinetics. These effects can narrow the processing window, which requires the careful optimization of extrusion and thermoforming parameters to prevent defects and ensure dimensional stability [6].

Thermoforming of bio-based and conventional composites reinforced with natural fillers has been explored across a range of different materials. In biodegradable systems, studies on cork-powder-reinforced PLA sheets demonstrated promising thermoformability while retaining biodegradability [7]. Bamboo-fabric-reinforced PLA composites have been evaluated for thermoforming, with results showing that the reinforcement influences the thermoformability behaviour of the sheets [8]. Morcillo Martín et al. incorporated agricultural by-products into bio-polyethylene for thermoformed food packaging applications, reporting enhanced processability, antioxidant activity, and mechanical performance [9].

Beyond biopolymers, several studies on petroleum-based matrices have provided valuable insights into the thermoforming behaviour of fibre-reinforced sheets. Wood flour–HDPE composites processed by vacuum forming showed satisfactory thermoforming rates at different wood contents under consistent conditions, though fibre distribution and stretching limitations affected uniformity [10]. Bhattacharyya et al. examined thermoformed wood-fibre/polypropylene composites, observing that fibre reduces the tendency of polypropylene to exhibit localized thinning [11]. In packaging-related applications, Afshariantorghabeh et al. demonstrated that plastic-coated fibre-based paperboards could be thermoformed into shallow shapes, though geometric complexity remained limited [12].

From a circular bioeconomy perspective, the valorisation of agro-industrial residues as functional fillers in biopolymer composites supports waste reduction and enables the production of fully compostable and disposable packaging materials [13]. Coffee silverskin (CSS), with its lignocellulosic profile, like other plant-based reinforcements, also contains proteins and antioxidants (such as caffeine, polyphenols, tannins, and melanoidins). These characteristics make CSS suitable to be used as a structural reinforcement material with antioxidant and thermal stabilizing properties in polymer matrices [14]. Petaloti et al. have demonstrated the potential of incorporating CSS into PLA matrices to develop sustainable biocomposites for food packaging applications, reporting improvements in stiffness and antioxidant activity [15]. However, there is a lack of studies correlating the thermoformability window of reinforced CSS biopolymer sheets, regarding the sheet thermoforming behaviour, processing conditions, and thermoformability limits.

The incorporation of coffee silverskin into PLA matrices has significant potential for developing sustainable materials. For thermoformed packaging applications, understanding the thermoforming response of the sheet, that is, its capacity to undergo controlled uniaxial and biaxial deformation, is essential to ensure processability and final part quality. The influence of CSS addition on thermoformability parameters such as draw ratio limits, processing temperature window, and deformation uniformity has not yet been comprehensively addressed. This study provides a detailed correlation between the thermoforming behaviour of CSS/PLA composites and their microstructural, thermal, and rheological characteristics, establishing a direct link between processing parameters and thermoforming behaviour.

Considering the above, this work aims to (i) assess the viability of producing thermoformable sheets from biopolymers reinforced with natural CSS fillers and (ii) characterize the influence of the CSS content on processing behaviour, microstructure, and mechanical performance. This study seeks to push the boundaries of research on the field of bio-based thermoformability sheets for compostable packaging and to foster the valorisation of coffee by-products through design in thermoforming. Moreover, the use of waste CSS into a biopolymer matrix aligns this work with sustainable development objectives, promoting reduced environmental impact and improved life-cycle performance compared to conventional fossil-based packaging.

## 2. Materials and Methods

### 2.1. Materials

In this work, the F38 biopolymer, extrusion grade, and injection grade supplied by Nurel S.A (Zaragoza, Spain) was used. F38 is a semi-crystalline PLA-based biopolymer, containing more than 70% renewable content and is 100% compostable. The extrusion grade (F38_ext_), with a Melt Volume-Flow Rate (MVR) of 1.8 cm^3^/10 min at 190 °C, 2.16 kg, was used for sheet extrusion and thermoforming, whereas the injection grade material (F38_inj_), with an MVR (190 °C, 2.16 kg) of 20 cm^3^/10 min, was used to produce the masterbatch. CSS was supplied by Meltino, a local Portuguese coffee roaster based in Braga, Portugal. Before preparing the composites, the CSS was sieved, to eliminate agglomerates and dried for at least two hours at 60 °C in a Binder forced convection oven. The CSS was used without any additional treatment to minimize the environmental and economic cost associated with these operations. The soluble fraction of CSS (13 wt.%) was determined using a Soxhlet extraction in ethanol.

### 2.2. Samples Preparation

#### 2.2.1. Masterbatch

The masterbatch was produced using a co-rotating Leistritz LSM 36/25D twin-screw extruder (Leistritz Extrusionstechnik GmbH, Nuremberg, Germany) with two gravimetric feeders, at 3 kg/h, to ensure accurate dosing of the components: 50 wt.% F38_inj_ and 50 wt.% CSS. The screws speed was set at 150 rpm and the temperature profile ranged from 120 °C, near the hopper, to 160 °C, at the extruder die. Prior to extrusion, all materials were dried for 3 h at 60 °C in a convection oven. The extruded composite was air cooled and then pelletized.

#### 2.2.2. Sheet Extrusion

The sheets were produced using a co-rotating Leistritz LSM 36/25D twin-screw extruder equipped with a flat die and a three-roll calender for calibration and cooling. The temperature profile of the extruder ranged from 120 °C near the hopper to 170 °C at the die. The screw speed was adjusted to 150 rpm, at a flow rate of 3 kg/h. The die lips and calender rolls were set to produce sheets with a thickness of 0.8 mm and the calender roll’s temperature at 70 °C. Prior to extrusion, the materials were dried for 3 h at 60 °C in a convection oven. The mass feed rates of the two gravimetric feeders were adjusted to achieve the target compositions shown in Table 1. The masterbatch was prepared using injection grade, selected for its lower melt viscosity, which enables the incorporation of a high filler content (50 wt.% CSS). For sheet extrusion, the masterbatch was subsequently blended with extrusion grade to provide higher melt strength and ensure proper sheet extrusion. The masterbatch was specifically used for the formulations containing CSS, whereas the compositions without CSS were prepared directly from the extrusion and injection grades of F38 in equivalent mass ratios to enable a consistent comparison between systems with and without filler. It is important to note that the quantities listed in Table 1 represent the nominal formulation ratios and larger batches were produced to provide sufficient material for all experimental analyses.

### 2.3. Structural and Morphological Characterization

The chemical bonding and molecular structure of materials were investigated using a Perkin-Elmer Spectrum 100 Fourier-transform infrared (FTIR) spectrometer fitted with an attenuated total reflection (ATR) detector (PerkinElmer, Waltham, MA, USA), equipped with a germanium crystal. FTIR spectra were collected in the range of 4000 and 600 cm^−1^ with 16 accumulations and a resolution of 4 cm^−1^.

Morphological analysis of F38/CSS composites was performed using a NanoSEM-FEI Nova 200 I Company, Hillsboro, OR, USA) scanning electron microscope. The samples were submerged and fractured under liquid nitrogen and coated with a thin film (2 nm) of Au-Pd (80–20 wt.%) in a high-resolution sputter coater, coupled to an MTM-20 Cressington High Resolution Thickness Controller (Cressington Scientific Instruments, Watford, UK). SEM analysis was performed on the sheets before and after thermoforming. Samples were collected from the centre of the sheet and were observed in the transverse direction of the extrusion. The thermoformed samples were collected from the region of the female mould, subjected to biaxial stretching. SEM analysis was performed on the sheets before and after thermoforming. Samples were collected from the centre of the sheet and were observed in the transverse direction of the extrusion. The thermoformed samples were collected from the region of the female mould, subjected to biaxial stretching. SEM analysis was performed on the sheets before and after thermoforming. Samples were collected from the centre of the sheet and were observed in the transverse direction of the extrusion. The thermoformed samples were collected from the region of the female mould, subjected to biaxial stretching.

### 2.4. Melt Flow Index

To assess the influence of CSS on the processability of the composites, the melt flow index was determined using Daventest equipment (Lenzing Instruments, Lenzing, Austria). The measurements were carried out under a 2.16 kg load at 180 °C. For each condition, at least three tests were performed, and the mean MFI values, together with their standard deviations, were plot.

### 2.5. Thermal Characterization

Thermogravimetric analysis (TGA) was carried out using a Q500 analyser (TA Instruments, New Castle, DE, USA) to evaluate the thermal degradation of CSS and its stability during the preparation of composites by extrusion. Samples with approximately 7 mg of CSS were placed in standard platinum crucibles and subjected to a heating programme from 40 °C to 600 °C at a heating rate of 10 °C/min under air atmosphere, simulating environmental degradation. To further assess CSS stability, the samples were heated from 30 °C to the target temperature at 10 °C/min under air atmosphere and kept at constant temperature for 36 min. This time corresponds to approximately 10 times the typical residence time of CSS inside the extruder. The tested isothermal temperatures ranged from 100 °C up to 260 °C.

Differential scanning calorimetry (DSC) was performed to evaluate the influence of filler addition on the crystallinity and glass transition of the composites. DSC of neat polymer and composite samples were conducted in two consecutive heating scans, to eliminate thermal history from processing. The tests were carried out using a Netzsch DSC 200 F3 Maia instrument (NETZSCH-Gerätebau GmbH, Selb, Germany), at a heating rate of 10 °C/min from 10 to 180 °C, under nitrogen flow. Sample mass (approx. 5 mg) was determined using a Perkin Elmer AD-4 micro-balance (PerkinElmer, Waltham, MA, USA).

### 2.6. Mechanical Characterization

Tensile properties were obtained at room temperature and a test speed of 10 mm/min, at a grip separation of 80 mm, according to ASTM D638 [16], using the universal test machine Instron 5969 equipped with an SVE 2 Non-Contacting Video Extensometer (Instron, Norwood, MA, USA). At least ten different specimens were tested and average values of tensile strength, elasticity modulus, and elongation at break were calculated. The tensile specimens were cut into the machine direction (MD) and transverse direction (TD) of the extrusion line using a standardized specimens cutter, according to ISO 527-4 [17]. The thickness of the specimens was measured in 3 points over the gauge length.

### 2.7. Optical Microscopy

Optical microscopy was used to evaluate the morphology and dispersion of CSS within the composites. Samples were taken from the centre of the extruded sheet along the extrusion direction. Cross-sections (15 microns thick) were cut using a Leica EM UC6 microtome (Leica Microsystems, Wetzlar, Germany) with a steel knife at room temperature and placed on a glass coverslip using Canada balsam. The samples were analyzed with an Olympus BH-2 optical microscope (Olympus Corporation, Tokyo, Japan) equipped with 4× and 10× lenses, in transmission mode.

### 2.8. Thermoforming Characterization

**Figure 1:**
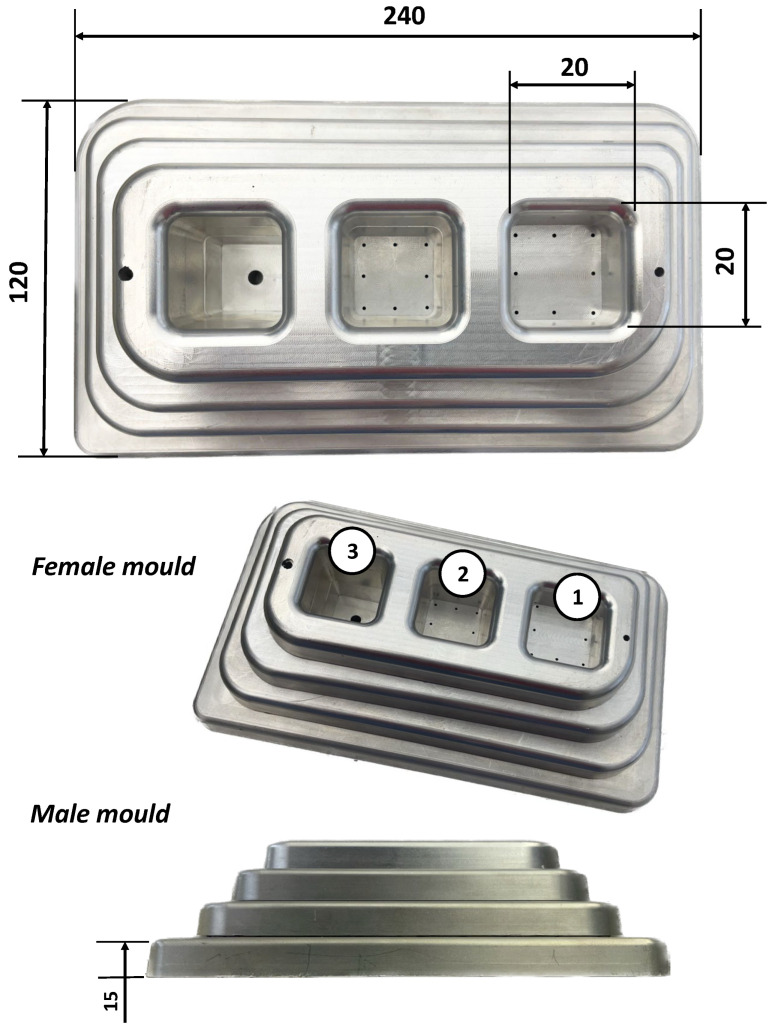
Thermoforming mould dimensions, in mm.

Thermoformability was evaluated by vacuum thermoforming tests, using an aluminum prototype mould (male and female), with different draw ratios. Figure 1 shows the prototype mould with the main dimensions. The female mould includes three cavities, 1, 2, and 3 (see Figure 1) with an approximate linear draw ratio (LDR) of 2, 4, and 6, and an approximate areal draw ratio (ADR) of 4, 7, and 10, respectively. Depending on the stretching capacity of the sheet, it is possible to reduce the depth of cavity 2 and 3 by using inserts to convert them with the same dimensions as cavity 1. The male mould comprises four plates with a height of 15 mm each, three of which are removable, according to the stretchability of the tested material. For a complete male mould (including all the 4 plates) the maximum LDR is approximately 1.5 and ADR of 4.5. The Formech 450DT thermoforming machine (Formech International Ltd., Harpenden, UK) was used with pre-cut sheets of 250 × 170 mm. Heating was applied manually by advancing and retracting the heating system, which is positioned above the sheet. The heating system consists of six quartz lamps, adjusted to a uniform power of 70% of their nominal value, ensuring a uniform temperature along the surface of the sheet. At the end of the heating time, the sheet temperature was recorded with an FLIR thermographic camera (Teledyne FLIR, Wilsonville, OR, USA) to record the thermoforming temperature for each composition. With the ThermaCAM Researcher Pro SR-2 software, a rectangle was drawn in the region of interest (centre of the sheet), and the average temperature was measured.

## 3. Results and Discussion

### 3.1. Structural and Morfological Analysis

To verify any structural changes or degradation between the CSS and the matrix caused by processing, the samples were evaluated using FTIR.

**Figure 2:**
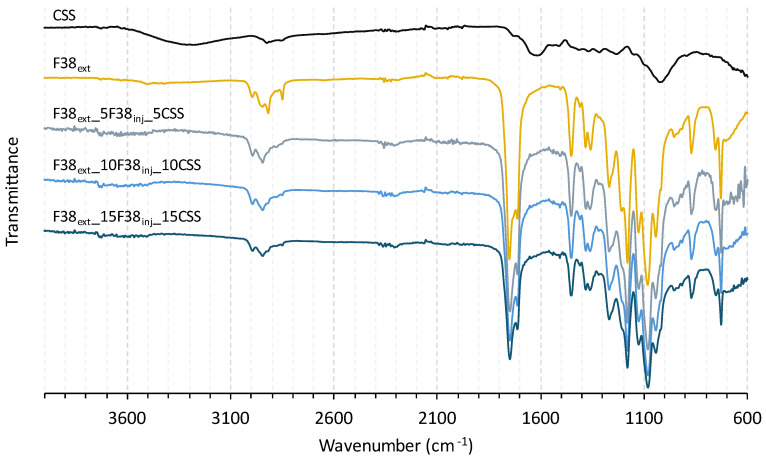
FTIR spectra of CSS, F38, and the CSS composites.

Figure 2 shows the FTIR spectra of CSS, F38 polymer, and the CSS composites. Analyzing the CSS spectrum, in general, the chemical groups of CSS’ constituents have the usual absorption bands of ligno-hemicellulosic materials [18,19]. The stretching of the hydroxyl group O-H, together with a modest contribution from N-H groups is primarily responsible for the presence of a broad band between 3600 and 3200 cm^−1^. The asymmetric and symmetric stretching of C-H bonds, abundant in lignin and hemicellulose, can be attributed to the two peaks at 2917 and 2843 cm^−1^, respectively. A smooth peak at 1730 cm^−1^ attributed to the C=O stretching of the acetyl group, characteristic of hemicellulose. The presence of a band at roughly 1620 cm^−1^ is characteristic of the carbonyl groups present in caffeine [20,21]. The typical aromatic ring vibrations of lignin appear between 1600 and 1480 cm^−1^. The wide band between 1100 and 890 cm^−1^ is associated with the cellulose C-O stretching and C-H rocking vibrations. The reported FTIR spectra of Arabica and Robusta roasted coffee samples, as well as those of Arabica green coffee samples, have already revealed similar bands [22,23]. Concerning the FTIR spectra of F38 polymers and CSS composites (F38_ext__5F38_inj__5CSS, F38_ext__10F38_inj__10CSS and F38_ext__15F38_inj__15CSS), it is possible to observe the main peaks corresponding to the PLA spectrum reported in the literature [24,25]. The peaks of relevance for F38 and the composites are about 1753 and 1184 cm^−1^, which belong to the C=O and the C–O–C stretching of PLA and the bands at 2918 cm^−1^ and 2848 cm^−1^ which are assigned to C-H stretching of -CH_3_. Moreover, increasing the coffee silverskin amount is visible; a broadening of the band around 1620 cm^−1^ due to the caffeine’s carbonyl groups present in CSS. Additionally, the absence of additional peaks to those of the matrix suggests that there was no significant degradation during processing.

### 3.2. Morphological Analysis

**Figure 3:**
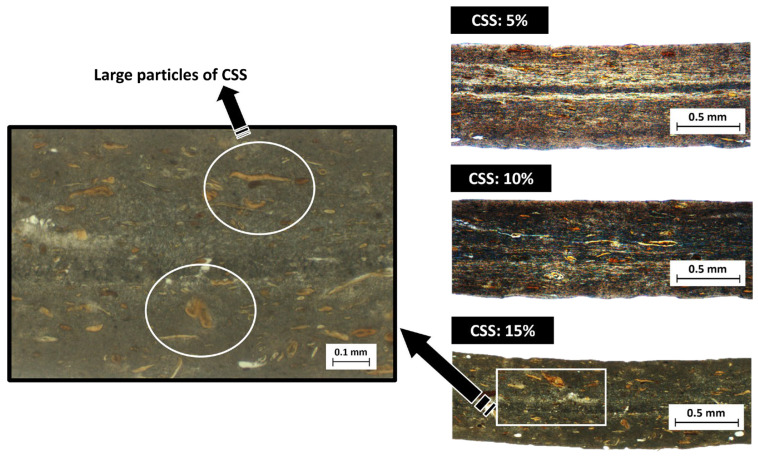
Microscopy images (bright field) of coffee silverskin composites.

Figure 3 shows optical microscopy images for all CSS composites. The images reveal CSS particles of different sizes and geometries heterogeneously distributed within the F38 matrix. The distributive mixing appears consistent across all samples, as the CSS particles are evenly dispersed throughout each specimen. The results indicate that the stresses and shear rates imposed during extrusion were sufficient to fragment fragile CSS particles, with most of them having dimensions smaller than 0.05 mm. In composites containing 5% CSS, no visible agglomeration is observed, whereas in composites with 15% CSS, the micrographs reveal the presence of some larger particles alongside a great number of smaller ones. The presence of larger CSS particles suggests that the dispersive mixing process within the extruder was not entirely effective under the employed mixing configuration. Therefore, to achieve a more homogeneous particle size distribution, a milling step prior to extrusion is recommended.

**Figure 4:**
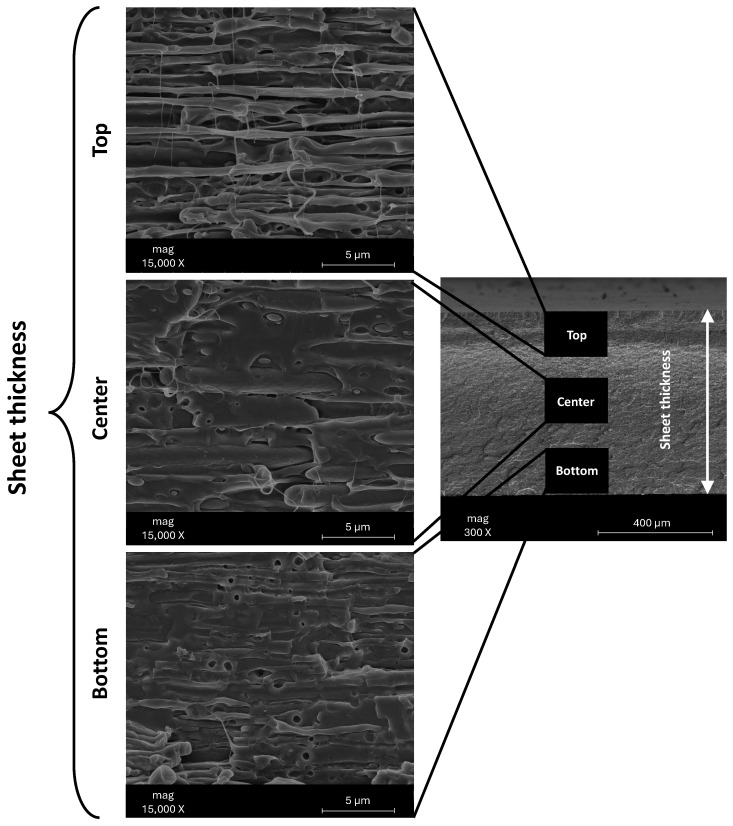
SEM images F38_ext_ across the sheet thickness and along the extrusion direction.

**Figure 5:**
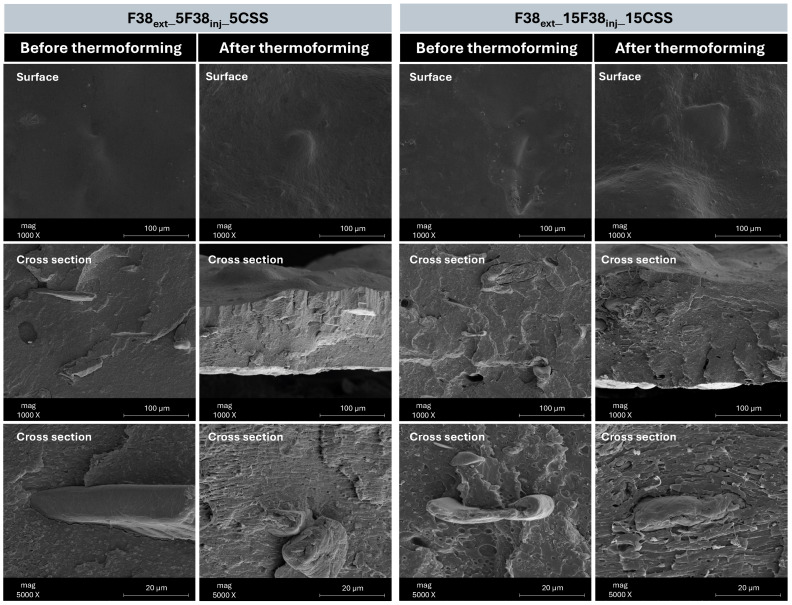
SEM images of composites F38_ext__5F38_inj__5CSS and F38_ext__15F38_inj__15CSS, before and after thermoforming.

Figure 4 shows the SEM images of the F38_ext_ across the sheet thickness, while Figure 5 of the composites with 5% and 15% of CSS before and after the thermoforming. Figure 5 includes a surface image of the sheet and a cross-section along the thickness. Figure 4 shows, as expected, the presence of two immiscible phases (PLA and thermoplastic starch, typical of this type of commercial blends) aligned along the flow direction that occurs during the sheet extrusion. In the top and the bottom, a higher orientation is observed than in the centre due to the higher shear rate.

In Figure 4, for the surface images both before and after thermoforming, it is observed that the CSS particles are well dispersed and surrounded by polymer. After thermoforming, the CSS particles become more visible on the surface due to the reduction in the sheet thickness that occurs upon stretching, thus increasing the surface roughness of the final product. Regarding the images along thickness, a good distribution of the CSS particles in the polymer matrix is observed, both for the compositions with 5% and 15% CSS. Before thermoforming, the fillers remain surrounded by the polymer, indicating fair filler/matrix interaction. However, the presence of voids (debonding mechanism) suggests limited interfacial interaction between matrix and CSS, which is more pronounced at higher concentration of CSS. After thermoforming, the CSS remains surrounded by the polymer, and the amount of debonding particles is reduced, indicating that stretching process may mechanically anchor the particles to the matrix.

### 3.3. Thermal Analysis

**Figure 6:**
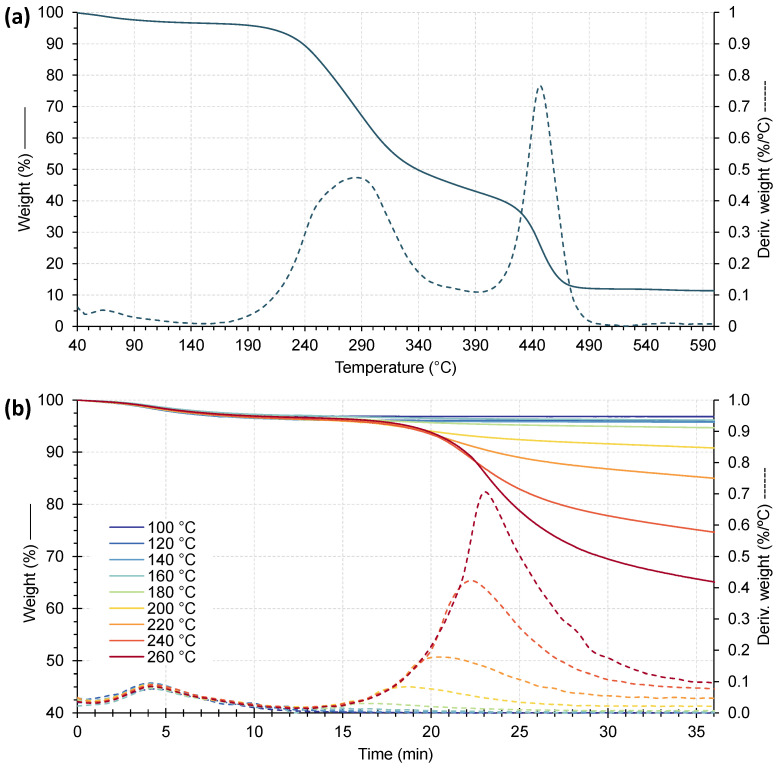
(**a**) TGA and DTG thermographs of CSS; (**b**) isothermal weight loss curves for CSS.

Figure 6 presents the weight loss of CSS (Figure 6a) and the isothermal weight loss curves (Figure 6b). The bold line represents the thermogravimetric analysis curve, whereas the dashed line corresponds to the derivative thermogravimetric (DTG) curve, whose peaks indicate the rate of temperature-dependent weight loss. In Figure 6a, a three-step thermal degradation is observed, defined as follows:

(i) The first region, corresponding to a weight loss of approximately 5 wt.%, occurs between the initial temperature and around 110 °C, and is attributed to moisture evaporation of the CSS [26].
(ii) The second region, observed between 200 °C and 340 °C, corresponds to the thermal decomposition of hemicellulose and cellulose, characterized by a pronounced weight loss of about 45 wt.% of the total mass loss. Similar results were reported by Kumar et al., where the main degradation stage was, likewise, attributed to the breakdown of hemicellulose and cellulose components [26].
(iii) The third region, corresponding to the gradual weight loss observed above 400 °C is attributed to the decomposition of lignin and proteins, as also reported by Gigante et al. [27].

Figure 6b presents the isothermal weight loss curves for CSS, including the initial heating phase up to the target temperature, followed by a 36 min isothermal stage. During the first 8 min of heating up to approximately 120 °C, all samples exhibit a weight loss of about 4 wt.%, primarily attributed to moisture evaporation. For the different isothermal curves, it is observed that up to 180 °C, the additional weight loss remains below 2 wt.%. The curve corresponding to 200 °C represents a transition point between minor and more pronounced weight loss. In the range of 220–260 °C, the mass loss after 36 min increases significantly, reaching approximately 15 wt.% and 33 wt.%, respectively. These results indicate that filler degradation is residual when processing temperatures do not exceed 180 °C. Processing at around 200 °C is still feasible, as long as the residence time within the extruder does not exceed 8–10 min. Therefore, maintaining the processing temperature below 200 °C ensures that CSS integrity is not significantly affected by thermal degradation, although some influence may arise from the formation of voids on the extruded sheet derived from the volatile release.

**Figure 7:**
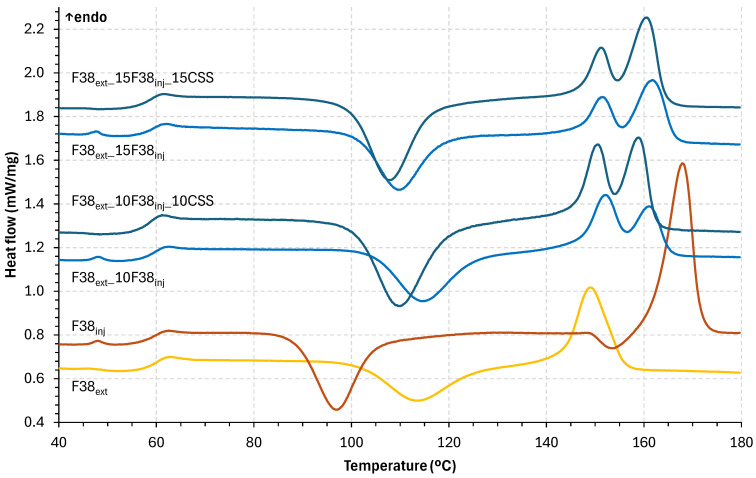
DSC of the second heating curves of F38 and CSS composites.

Figure 7 and Table 2 show the DSC results for the F38 grades and CSS composites (for 10 and 15 g of CSS). The graphs clearly show the materials’ glass transition (Tg), cold crystallization (Tc), and melting (Tm). Despite sharing the same commercial name, both F38 grades show distinct thermal behaviours. F38_inj_ exhibits a small melting peak at 48 °C, likely associated with the presence of processing aid used to increase material fluidity, and a Tg at 60 °C. In CSS composites, this peak is not observed, likely due to its low intensity falling below the detection threshold of the DSC technique, which does not necessarily indicate the absence of the phase. Also, F38_inj_ has lower Tc (97 °C) and higher Tm (168 °C, with a secondary Tc associated at 154 °C) than F38_ext_, showing that the grade formulation impacts the thermal behaviour of the PLA matrix. The secondary Tc observed for F38_inj_ (around 155 °C) can be related to the formation of a different crystalline structure (transformation from α’ to α phase) during second heating, as reported for PLA in the literature [28]. For neat PLA, rapid cooling can originate the development of less perfect and disorganized crystalline structures. In the formulations containing CSS, this secondary Tc is not detected, likely because CSS acts as a nucleating agent that promotes a more uniform crystallization, reducing the occurrence or detectability of the secondary crystalline phase under the applied DSC conditions [29]. This suggests that F38_ext_ is less affected by cooling rate and less affected by cold crystallization phenomena, reducing the risk of phase transformation at lower temperatures. On the other hand, F38_inj_ crystals are more stable as indicated by the higher energy required for their melting. This behaviour was also seen in other bio-based polymers [30,31]. Blending the F38 grades show no change on Tg but shift Tc and Tm for intermedium values, reflecting miscibility between both neat grades. As the F38_inj_ content increases, the intensity of the secondary melting peak becomes more pronounced, and the melting temperature shifts toward lower values, closer to that of the F38_inj_ grade. For the CSS composites, there was no change in the F38’s glass transition temperature. Although the amorphous phase is not affected, the present of CSS particles lowers Tc, indicating that CSS acts as a nucleating agent for crystal growth. Moreover, CSS appears to promote the formation of the more stable crystalline phase, as evidenced by the increased intensity of the second melting peak, which is particularly noticeable in the F38_ext__10F38_inj__10CSS composite. This phenomenon has occurred in other investigations with both coffee silverskin [19] and other natural fibres [25]. It was observed that the melting temperature of composites varied slightly from that of virgin polymers, shifting to lower values by around 2 and 8 °C. The addition of CSS in the polymer matrix was responsible for this variance. Yet, this fluctuation was not statistically significant, indicating that CSS addition had no adverse effects on the processing temperature of composites.

### 3.4. Melt Flow Index

**Figure 8:**
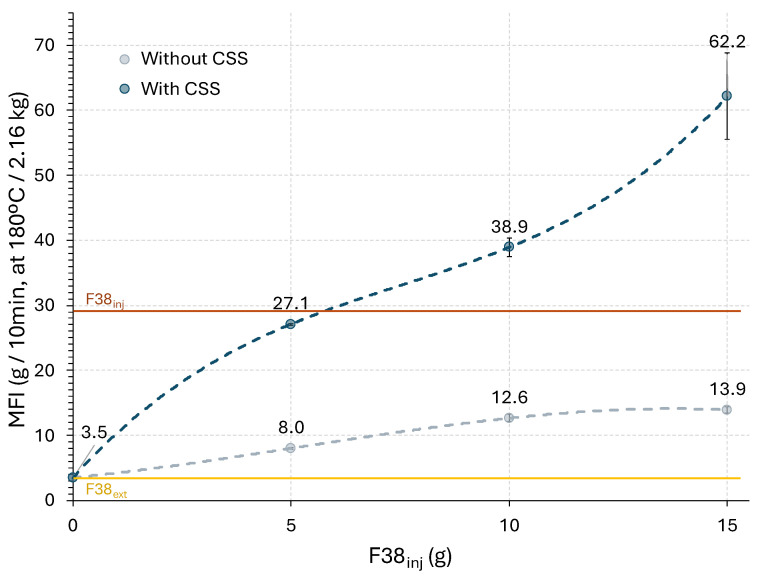
Melt flow index of F38 virgin blends and their CSS-containing counterparts.

The melt flow index (MFI) of virgin F38 blends and their CSS-containing counterparts is presented in Figure 8. For compositions containing CSS, the data are presented as a function of the relative proportion of F38 in the blends (Table 1), allowing a clear comparison between the different formulations. Although the extrusion-grade F38 melts at approximately 150 °C, the injection-grade F38 requires 175–180 °C for complete melting. Thus, MFI tests were performed at 180 °C to ensure homogeneous melting of both polymers and reliable measurement of melt flow. As expected, increasing the proportion of injection-grade F38 results in a significant rise in MFI, due to its lower average molecular weight and higher melt fluidity compared to the extrusion grade. While extrusion-grade F38 exhibits a low MFI of 3.5 g/10 min, the injection-grade polymer reaches values close to 29 g/10 min at 180 °C, confirming the differences in rheological behaviour between the two commercial grades. The most notable outcome, however, is observed when comparing the neat blends with their CSS-reinforced counterparts. Contrary to the behaviour usually reported for polymer composites with natural fibres (where viscosity increases due to the restriction of polymer chain mobility [32]) the addition of CSS led to a sharp rise in MFI, reaching 27.1, 38.9, and even 62.2 g/10 min for composites containing 5, 10, and 15% CSS, respectively. Several mechanisms may contribute to this atypical behaviour. Coffee residues such as CSS contain small amounts of oils and lipids (mainly triacylglycerols), which can act as internal lubricants or plasticizers, reducing intermolecular forces within the polymer matrix and enhancing chain mobility [33]. As determined by Soxhlet extraction in ethanol, CSS contains up to 13 wt.% of soluble fractions. In addition, the proteins naturally present in CSS may also provide a plasticizing effect, as reported by Kumar et al. [26]. This plasticizing effect is defined as the ability of substances to decrease the strength of intermolecular forces among polymer chains, thereby enhancing their mobility and decreasing the overall viscosity. Another possible explanation for the observed MFI increase is hydrolytic degradation of PLA during melt processing. PLA is particularly sensitive to hydrolysis, where residual moisture promotes cleavage of ester bonds, lowering the molecular weight, and enhancing melt flow. Several factors, including temperature, pH, water concentration, and the presence of catalysts, can influence the rate of this reaction [34,35]. Although the CSS was dried prior to compounding, its lignocellulosic structure may have retained some residual moisture that could not be fully removed. At extrusion and testing temperatures, this residual moisture may have accelerated hydrolytic chain scission, leading to higher MFI values. Similar effects have been consistently reported in composites containing lignocellulosic fillers, where even small amounts of moisture can significantly intensify hydrolysis during processing [14,36]. In addition to the plasticization and moisture-induced hydrolysis discussed above, a chemical contribution from the lignocellulosic structure of CSS cannot be excluded. The hydroxyl groups of cellulose and hemicellulose, in the presence of residual water and processing heat, can interact with the ester linkages of PLA, promoting hydrolytic cleavage of the polymer chains [37,38].

### 3.5. Mechanical Properties

**Figure 9:**
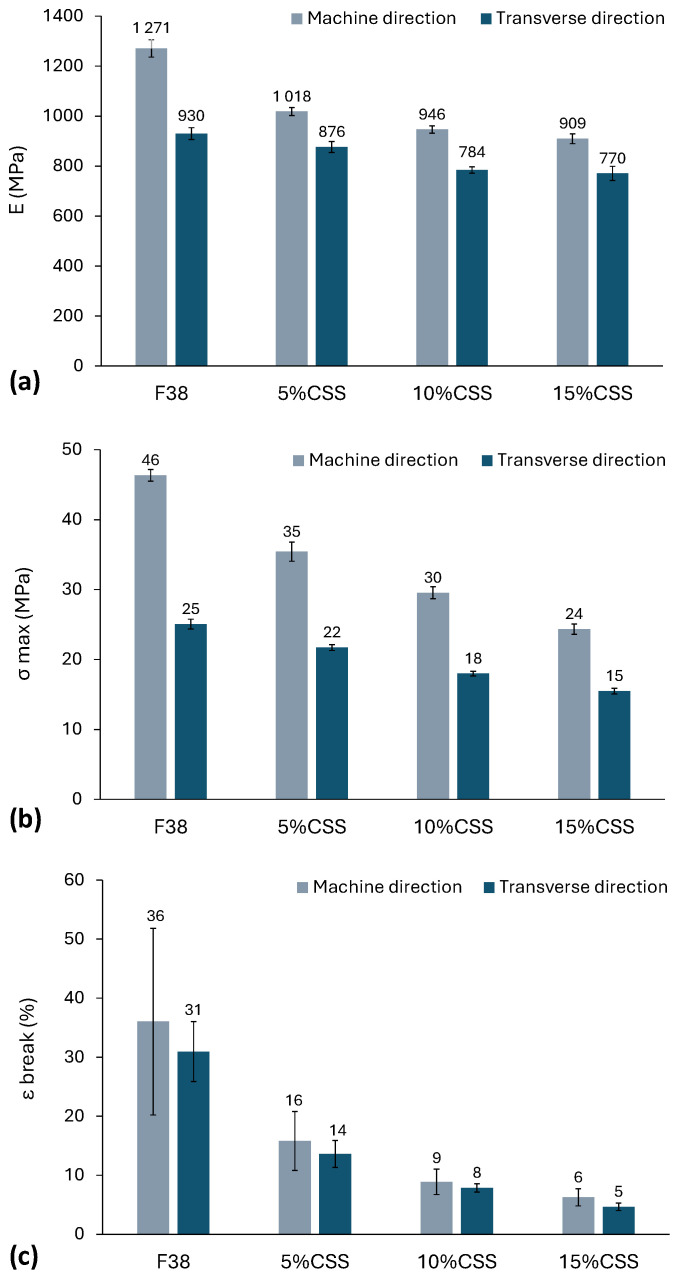
Tensile properties of F38 and the coffee silverskin composites sheets, prior to thermoforming: (**a**) Young’s modulus, (**b**) tensile strength, and (**c**) deformation at break.

Figure 9 presents the mechanical properties of neat F38_ext_ and CSS composites, at the machine and transverse direction. Figure 9a presents the Young’s modulus, Figure 9b the tensile strength, and Figure 9c the strain at break. The high standard deviation observed in the deformation at break for F38 suggests that, at the tested thickness, the material is near the transition from plane stress (ductile fracture, typical of thin samples) to plane strain (brittle fracture, typical of thick samples) [39]. The addition of filler shows an inverse relationship between mechanical properties and CSS content. Furthermore, the mechanical strength is greater in the machine direction than in the transverse direction, primarily due to molecular orientation effects. In the extrusion direction, and for the maximum of 15% CSS composites, the Young’s Modulus, the tensile strength, and the elongation at break of the composite decreased by 28%, 48% and 83%, respectively, when compared to the neat polymer. For the transverse direction, the decreasing is lower, being 17%, 40% and 85% for Young’s Modulus, tensile strength, and the elongation at break, respectively. According to the literature, adding CSS to different biopolymers materials increases the young’s modulus [40,41]. There have been reports of both an increase [40] and a decrease [42,43] in mechanical properties, depending on the nature of the filler and its interaction with the matrix. Enhanced tensile strength is typically associated with the chemical modification of the natural filler, which improves the interfacial adhesion between both phases. A decrease in elongation at break is a usual behaviour in the properties of thermoplastic composites, according to published research [40,41,44]. This behaviour is mainly attributed to the lack of full compatibilization between the polymer matrix and the filler (as shown in SEM analysis), which results in interfacial voids and poor stress transfer efficiency, ultimately diminishing the overall mechanical performance. Similar findings were reported in other research works [32,45,46,47]. Another explanation may arise from the matrix degradation and decrease in the molecular weight, due to thermo-mechanical hydrolysis upon compounding. As suggested by the MFI results, which showed a substantial increase in flowability for the CSS composites, residual moisture may have promoted the hydrolysis of PLA chains, leading to a decrease in molecular mass and, ultimately, a deterioration of the mechanical properties.

### 3.6. Thermoformability

**Figure 10:**
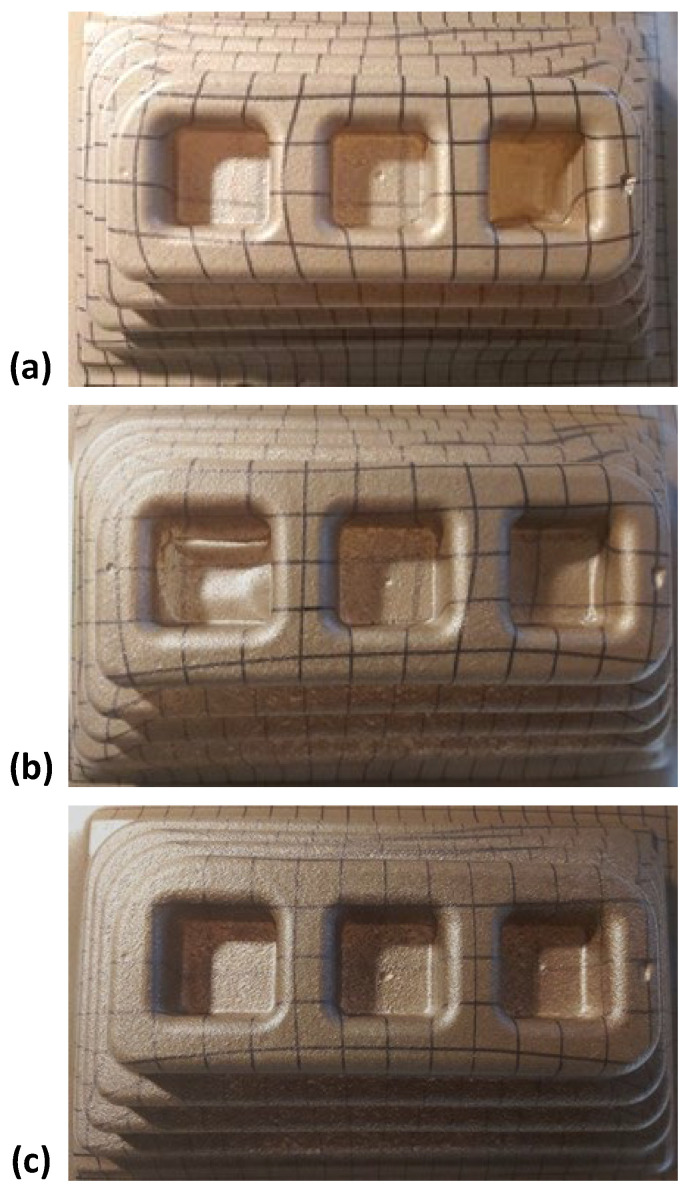
Thermoformed parts with (**a**) F38_ext__5F38_inj__5CSS, (**b**) F38_ext__10F38_inj__10CSS, and (**c**) F38_ext__15F38_inj__15CSS.

In order to construct the thermoformability processing window, the extruded sheets were thermoformed at different temperatures, recording the minimum and maximum temperature that allowed a good replication of the mould without any visible defects. Figure 10 shows an example of well-thermoformed parts of CSS composites with 5% CSS, 10% CSS, and 15% CSS at 150 °C, 145 °C, and 135 °C, respectively. For all compositions, and at least for one sheet temperature, thermoforming was able to reproduce all levels of the male mould and of the female mould. The results also show that in the region corresponding to the male mould, the deformation of the sheet is essentially uniaxial, and that at the base of the female mould, it is biaxial. By analyzing the deformation of the previously drawn squares, it can also be concluded that there is local deformation greater than 2 (LDR > 2) in the male mould and biaxial deformation greater than 4 (LDR > 4) at the base of the female mould. It can also be observed that, even for composites with 15% CSS, the material retains its deformation ability, obtaining parts with adequate replica of the mould contours.

**Figure 11:**
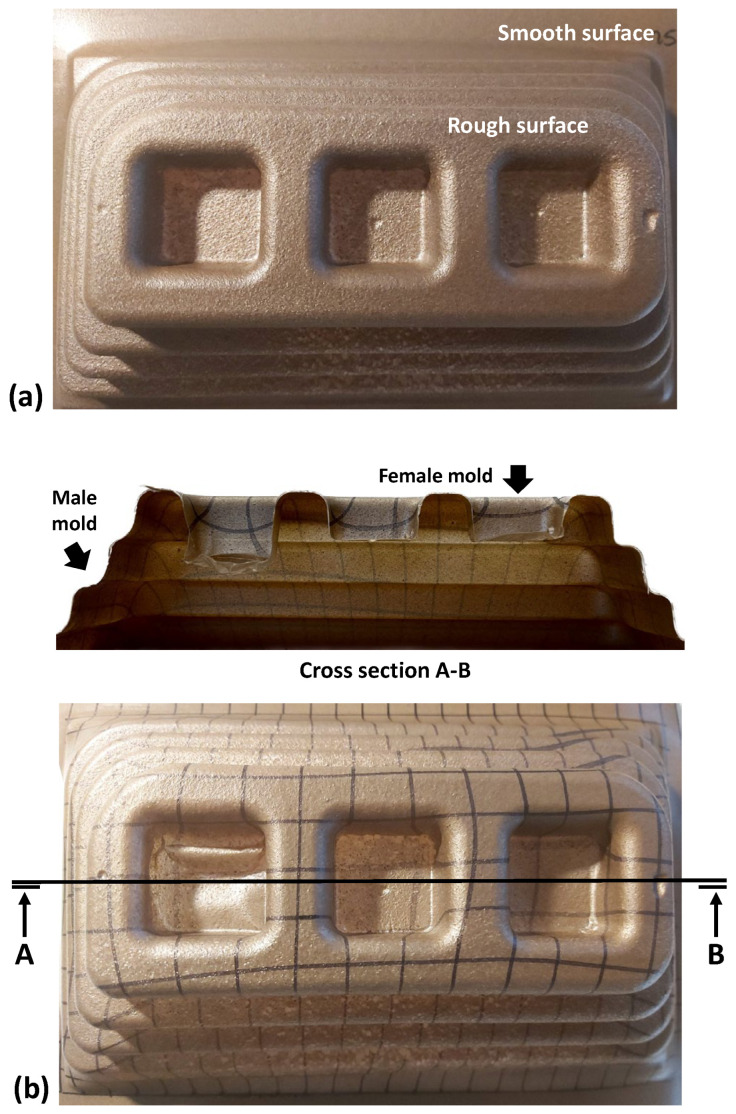
(**a**) Surface aesthetics: thermoformed extruded sheet region; (**b**) cross-section of the thermoformed part. Note: in this thermoforming test, the female mould includes only two cavity depths.

Figure 11a shows, in more detail, the difference between the surface aesthetics of the extruded sheet and the thermoformed region. Due to the heating and sheet stretching during thermoforming, the sheet thickness is reduced, and CSS morphology is revealed at the part surface (since CSS does not deform). Despite being more noticeable, the CSS particles are still surrounded by the F38 matrix, remaining fixed to the sheet. As a practical consequence, the surface roughness increases, making the CSS fillers perceptible to touch. Figure 11b shows a cross-section of the thermoformed part. It is possible to observe that the part replicates the contours of the mould, both in the male mould region and in the female mould region. In the female mould, a hole is formed with an LDR = 4. Finally, in the cutting section, it is possible to observe a homogeneous distribution of CSS fillers across the entire area of the sheet.

**Figure 12:**
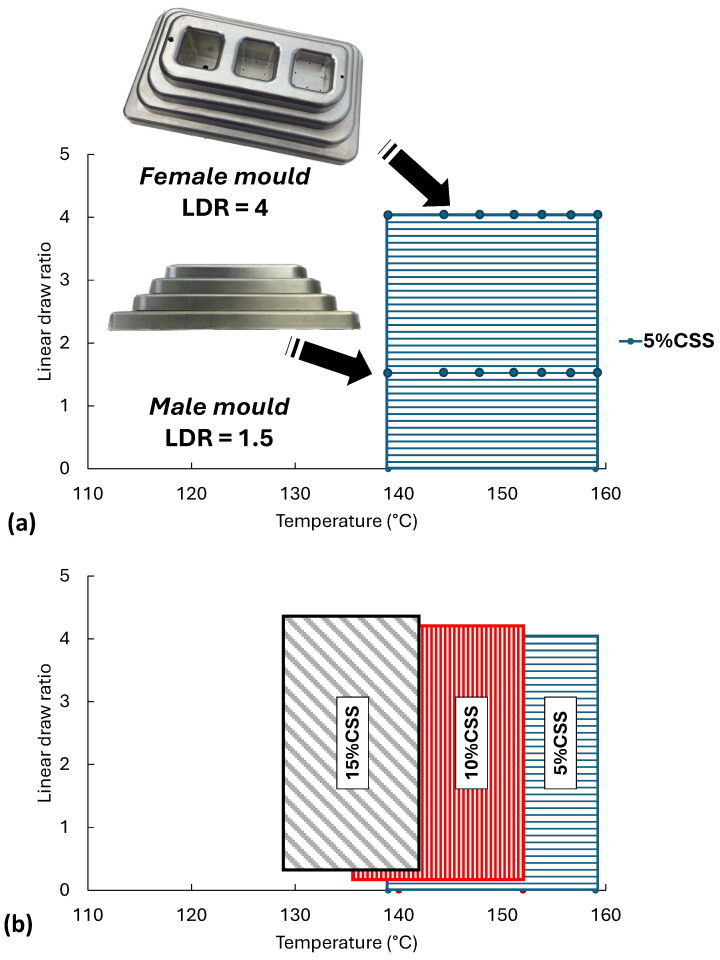
Thermoformability of the developed composite materials: (**a**) adopted procedure; (**b**) thermoformability range.

Figure 12 shows the thermoformability processing windows of the extruded composite sheets. Figure 12a depicted the adopted procedure using F38_ext__5F38_inj__5CSS composite as example, while Figure 12b shows the results. The procedure adopted was as follows: (i) Heating the sheet until it reaches a minimum temperature of T1. (ii) Test thermoformability, i.e., evaluate the ability to replicate the mould cavities; if the mould contours were accurately replicated, the temperature T1 was considered the minimum thermoforming temperature. Otherwise, the temperature was increased until a value that enables obtaining a complete part was found. That temperature was than recorded and designated as the minimum thermoforming. (iii) Increase the sheet temperature and repeat the step (ii) of this procedure. (iv) Continuously increase the sheet temperature until defects attributable to excessive temperature, such as sheet rupture during the thermoforming process, were observed. Record the previous temperature value as the maximum thermoforming temperature. The deformation range presented was derived from visual inspection of the thermoformed parts and from the deformation pattern of the pre-marked grids on the sheet surface, based on the known mould geometry. Following this methodology, it was possible to obtain parts with all levels of the male mould, maximum LDR = 1.6, and only depths 1 and 2 for the female mould, corresponding to the maximum LDR = 4. Thus, the dots represent the experimental results, while the shaded area in blue represents all the temperature/LDR combinations for which it will be possible (but not tested) to obtain thermoformed parts. The procedure was adopted for the other composites, and the results are presented in Figure 12b. The red shaded area corresponds to composites with 10% CSS while the black shaded area corresponds to composites with 15% CSS. For 10% CSS and 15% CSS, there is a shift on the YY axis to make it possible to observe the limits of all shaded areas. For composite materials with 5% CSS, the minimum recorded thermoforming temperature was 138 °C while the maximum was 158 °C; for 10% of CSS, the minimum temperature was 136 °C and the maximum was 153 °C, while for materials with 15% CSS, the minimum temperature was 129 °C and the maximum was 142 °C. These observed limit values correspond to temperature ranges of 21 °C, 17 °C, and 13 °C for composites with 5%, 10%, and 15% CSS, respectively. In practice, this means that as the amount of filler increases, controlling the sheet temperature becomes more and more demanding to successfully obtain thermoformed parts.

In addition to the reduction in the range of the operating temperature window increasing filler content, the results also show that the operating window shifts to the left, towards the lower sheet temperature. These results are consistent with those observed for MFI, where it is found that increasing CSS decreases the viscosity of the composites. This has a negative impact on the extensional viscosity of the matrix, which decreases its ability to biaxially deform during thermoforming. Increasing the amount of CSS also contributes to a lower ability to deform composite materials within the thermoforming temperature window.

## 4. Conclusions

Coffee silverskin, a by-product of coffee processing, was successfully incorporated into PLA-based formulations at concentrations up to 15 wt.%, reducing the overall content of the biopolymer matrix. The key findings are summarized as follows:

- DSC analysis indicated that the incorporation of CSS fillers did not affect the glass transition temperature of the PLA matrix but caused a slight decrease in the cold crystallization temperature, indicating a nucleating effect that promotes crystal growth. The melting temperature showed only minor, statistically insignificant variations, suggesting that CSS addition does not adversely impact the processing temperature of the composites.
- In all the resulting biopolymer blends, the elastic modulus, tensile strength, and elongation at break were all reduced. This behaviour is likely due to factors such as the irregular particle size of the filler, the absence of a compatibilizer agent, and differences in chemical structures and polarity between the filler and the polymer matrix.
- SEM observations confirmed the presence of CSS particles within the PLA matrices. The polymer fibres appeared well interwoven and relatively uniform. Compared to the neat polymer, the addition of the filler tends to moderate the blend fluidity while maintaining sufficient deformation resistance, making it possible to thermoform all compositions.

Overall, the mechanical properties of the composites are reasonable and can be chosen to provide various features depending on the additives employed. Additionally, the materials show potential for further studies intended to establish the composting and disposal profile to have a favourable environmental impact.

These findings demonstrate that the incorporation of coffee silverskin in a biopolymer matrix represents an effective waste valorisation strategy while reducing the consumption of virgin materials. This approach provides a sustainable opportunity, due to its favourable economic and environmental implications.

Future research will focus on evaluating the compostability and environmental performance of the developed thermoformed materials under controlled degradation conditions.

## Figures and Tables

**Table 1:** Formulations of the composites.

	F38_ext_ (g)	F38_inj_ (g)	CSS (g)
F38_ext__5F38_inj_	90	5	-
F38_ext__5F38_inj__5CSS	90	5	5
F38_ext__10F38_inj_	80	10	-
F38_ext__10F38_inj__10CSS	80	10	10
F38_ext__15F38_inj_	70	15	-
F38_ext__15F38_inj__15CSS	70	15	15

**Table 2:** DSC analysis of neat F38 and CSS composites *.

Samples	Tg (°C)	Tc (°C)	Tm (°C)
F38_ext_	61	114	149
F38_inj_	59	97/154	168
F38_ext__10F38_inj_	60	115	152/161
F38_ext__10F38_inj__10CSS	59	110	150/159
F38_ext__15F38_inj_	60	110	151/162
F38_ext__15F38_inj__15CSS	59	108	151/161

* Standard deviation is below 5% of the mean value.

## Data Availability

The original contributions presented in this study are included in the article. Further inquiries can be directed to the corresponding author.

## References

[1] Valdés A., Mellinas A.C., Ramos M., Garrigós M.C., Jiménez A. (2014). Natural Additives and Agricultural Wastes in Biopolymer Formulations for Food Packaging. Front. Chem..

[2] Reichert C.L., Bugnicourt E., Coltelli M.B., Cinelli P., Lazzeri A., Canesi I., Braca F., Martínez B.M., Alonso R., Agostinis L. (2020). Bio-Based Packaging: Materials, Modifications, Industrial Applications and Sustainability. Polymers.

[3] Ahmad M.N., Ishak M.R., Taha M.M., Mustapha F., Leman Z. (2022). Rheological Properties of Natural Fiber Reinforced Thermoplastic Composite for Fused Deposition Modeling (FDM): A Short Review. J. Adv. Res. Fluid Mech. Therm. Sci..

[4] Pulikkalparambil H., Varghese S.A., Chonhenchob V., Nampitch T., Jarupan L., Harnkarnsujarit N. (2023). Recent Advances in Natural Fibre-Based Materials for Food Packaging Applications. Polymers.

[5] Kabir M.M., Wang H., Lau K.T., Cardona F. (2012). Chemical Treatments on Plant-Based Natural Fibre Reinforced Polymer Composites: An Overview. Compos. B Eng..

[6] McKay I., Vargas J., Yang L., Felfel R.M. (2024). A Review of Natural Fibres and Biopolymer Composites: Progress, Limitations, and Enhancement Strategies. Materials.

[7] Moutinho L.G., Soares E., Oliveira M. (2025). Thermoforming of Bio-Based Polylactic Acid (PLA) Sheets Reinforced with Cork Powder. Mater. Today Commun..

[8] Nurul Fazita M.R., Jayaraman K., Bhattacharyya D. (2016). Formability Analysis of Bamboo Fabric Reinforced Poly (Lactic) Acid Composites. Materials.

[9] Morcillo-Martín R., Rabasco-Vílchez L., Jiménez-Jiménez F., Espinosa E., Tarrés Q., Rodríguez A. (2025). Thermoformed Fiber-Polyethylene Biocomposites: A Circular Food Packaging on Cherry Tomatoes. Food Bioproc. Technol..

[10] Afshariantorghabeh S., Kärki T., Leminen V. (2023). Thermoformability Study of Wood Flour–HDPE Composites with Variations in Wood Content Under Vacuum Forming. Heliyon.

[11] Bhattacharyya D., Bowis M., Jayaraman K. (2003). Thermoforming Woodfibre-Polypropylene Composite Sheets. Compos. Sci. Technol..

[12] Afshariantorghabeh S., Kärki T., Leminen V. (2022). Three-Dimensional Forming of Plastic-Coated Fibre-Based Materials Using a Thermoforming Process. Packag. Technol. Sci..

[13] Mishra B., Mohanta Y.K., Reddy C.N., Reddy S.D.M., Mandal S.K., Yadavalli R., Sarma H. (2023). Valorization of Agro-Industrial Biowaste to Biomaterials: An Innovative Circular Bioeconomy Approach. Circ. Econ..

[14] Toschi T.G., Cardenia V., Bonaga G., Mandrioli M., Rodriguez-Estrada M.T. (2014). Coffee Silverskin: Characterization, Possible Uses, and Safety Aspects. J. Agric. Food Chem..

[15] Petaloti A.I., Achilias D.S. (2024). The Development of Sustainable Biocomposite Materials Based on Poly(Lactic Acid) and Silverskin, a Coffee Industry By-Product, for Food Packaging Applications. Sustainability.

[16] (2022). Standard Test Method for Tensile Properties of Plastics.

[17] (2023). Plastics—Determination of Tensile Properties Part 4: Test Conditions for Isotropic and Orthotropic Fibre-Reinforced Plastic Composites.

[18] Ballesteros L.F., Teixeira J.A., Mussatto S.I. (2014). Chemical, Functional, and Structural Properties of Spent Coffee Grounds and Coffee Silverskin. Food Bioproc. Technol..

[19] Sarasini F., Tirillò J., Zuorro A., Maffei G., Lavecchia R., Puglia D., Dominici F., Luzi F., Valente T., Torre L. (2018). Recycling Coffee Silverskin in Sustainable Composites Based on a Poly(Butylene Adipate-Co-Terephthalate)/Poly(3-Hydroxybutyrate-Co-3-Hydroxyvalerate) Matrix. Ind. Crops Prod..

[20] Ribeiro J.S., Salva T.J., Ferreira M.M.C. (2010). Chemometric Studies for Quality Control of Processed Brazilian Coffees Using Drifts. J. Food Qual..

[21] Wang J., Jun S., Bittenbender H.C., Gautz L., Li Q.X. (2009). Fourier Transform Infrared Spectroscopy for Kona Coffee Authentication. J. Food Sci..

[22] Craig A.P., Franca A.S., Oliveira L.S. (2012). Evaluation of the Potential of FTIR and Chemometrics for Separation between Defective and Non-Defective Coffees. Food Chem..

[23] Kemsley E.K., Ruault S., Wilson R.H. (1995). Analytical, Nutritional and Clinical Methods Section Discrimination Between Co&a Arabica and Co&a Canephora Variant Robusta Beans Using Infrared Spectroscopy. Food Chem..

[24] Wu C.S., Liao H.T. (2005). A New Biodegradable Blends Prepared from Polylactide and Hyaluronic Acid. Polymer.

[25] Mofokeng J.P., Luyt A.S., Tábi T., Kovács J. (2012). Comparison of Injection Moulded, Natural Fibre-Reinforced Composites with PP and PLA as Matrices. J. Thermoplast. Compos. Mater..

[26] Kumar K.S., Gairola S., Singh I. (2024). Waste Coffee Silverskin as a Potential Filler in Sustainable Composites: Mechanical, Thermal, and Microstructural Analysis. Ind. Crops Prod..

[27] Gigante V., Seggiani M., Cinelli P., Signori F., Vania A., Navarini L., Amato G., Lazzeri A. (2021). Utilization of Coffee Silverskin in the Production of Poly(3-Hydroxybutyrate-Co-3-Hydroxyvalerate) Biopolymer-Based Thermoplastic Biocomposites for Food Contact Applications. Compos. Part A Appl. Sci. Manuf..

[28] Colaers M., Thielemans W., Goderis B. (2025). Quantitative DSC Assessment of the Polymorph-Specific Crystallinity of Poly(Lactic Acid) and the Impact of a Self-Assembling Nucleating Agent and PEG Plasticizer. Polymers.

[29] Novák J., Běhálek L., Borůvka M., Lenfeld P. (2022). The Physical Properties and Crystallization Kinetics of Biocomposite Films Based on PLLA and Spent Coffee Grounds. Materials.

[30] Arrieta M.P., López J., Ferrándiz S., Peltzer M.A. (2013). Characterization of PLA-Limonene Blends for Food Packaging Applications. Polym. Test..

[31] Luzi F., Fortunati E., Jiménez A., Puglia D., Pezzolla D., Gigliotti G., Kenny J.M., Chiralt A., Torre L. (2016). Production and Characterization of PLA_PBS Biodegradable Blends Reinforced with Cellulose Nanocrystals Extracted from Hemp Fibres. Ind. Crops Prod..

[32] Huang L., Mu B., Yi X., Li S., Wang Q. (2018). Sustainable Use of Coffee Husks For Reinforcing Polyethylene Composites. J. Polym. Environ..

[33] Speer K., Kölling-Speer I. (2006). The Lipid Fraction of the Coffee Bean. Braz. J. Plant Physiol..

[34] Siparsky G.L., Voorhees K.J., Miao F. (1998). Hydrolysis of Polylactic Acid (PLA) and Polycaprolactone (PCL) in Aqueous Acetonitrile Solutions: Autocatalysis. J. Environ. Polym. Degrad..

[35] Elsawy M.A., Kim K.H., Park J.W., Deep A. (2017). Hydrolytic Degradation of Polylactic Acid (PLA) and Its Composites. Renew. Sustain. Energy Rev..

[36] Li X., Lin Y., Liu M., Meng L., Li C. (2023). A Review of Research and Application of Polylactic Acid Composites. J. Appl. Polym. Sci..

[37] Wan Ishak W.H., Rosli N.A., Ahmad I. (2020). Influence of Amorphous Cellulose on Mechanical, Thermal, and Hydrolytic Degradation of Poly(Lactic Acid) Biocomposites. Sci. Rep..

[38] Momeni S., Craplewe K., Safder M., Luz S., Sauvageau D., Elias A. (2023). Accelerating the Biodegradation of Poly(Lactic Acid) through the Inclusion of Plant Fibers: A Review of Recent Advances. ACS Sustain. Chem. Eng..

[39] Xu S., Tahon J.F., De-Waele I., Stoclet G., Gaucher V. (2020). Brittle-to-Ductile Transition of Pla Induced by Macromolecular Orientation. Express Polym. Lett..

[40] Sarasini F., Luzi F., Dominici F., Maffei G., Iannone A., Zuorro A., Lavecchia R., Torre L., Carbonell-Verdu A., Balart R. (2018). Effect of Different Compatibilizers on Sustainable Composites Based on a PHBV/PBAT Matrix Filled with Coffee Silverskin. Polymers.

[41] Ghazvini A.K.A., Ormondroyd G., Curling S., Saccani A., Sisti L. (2022). An Investigation on the Possible Use of Coffee Silverskin in PLA/PBS Composites. J. Appl. Polym. Sci..

[42] Moustafa H., Guizani C., Dufresne A. (2016). Sustainable Biodegradable Coffee Grounds Filler and Its Effect on the Hydrophobicity, Mechanical and Thermal Properties of Biodegradable PBAT Composites. J. Appl. Polym. Sci..

[43] Quiles-Carrillo L., Montanes N., Garcia-Garcia D., Carbonell-Verdu A., Balart R., Torres-Giner S. (2018). Effect of Different Compatibilizers on Injection-Molded Green Composite Pieces Based on Polylactide Filled with Almond Shell Flour. Compos. B Eng..

[44] Vannini M., Marchese P., Sisti L., Saccani A., Mu T., Sun H., Celli A. (2021). Integrated Efforts for the Valorization of Sweet Potato By-Products within a Circular Economy Concept: Biocomposites for Packaging Applications Close the Loop. Polymers.

[45] Suaduang N., Ross S., Ross G.M., Pratumshat S., Mahasaranon S. (2019). Effect of Spent Coffee Grounds Filler on the Physical and Mechanical Properties of Poly(Lactic Acid) Bio-Composite Films. Mater. Today Proc..

[46] Yang H.S., Wolcott M.P., Kim H.S., Kim S., Kim H.J. (2007). Effect of Different Compatibilizing Agents on the Mechanical Properties of Lignocellulosic Material Filled Polyethylene Bio-Composites. Compos. Struct..

[47] Zhang Q., Li Y., Cai H., Lin X., Yi W., Zhang J. (2019). Properties Comparison of High Density Polyethylene Composites Filled with Three Kinds of Shell Fibers. Results Phys..

